# Associations between polygenic risk score and covid-19 susceptibility and severity across ethnic groups: UK Biobank analysis

**DOI:** 10.1186/s12920-023-01584-x

**Published:** 2023-06-30

**Authors:** Raabia Farooqi, Jaspal S. Kooner, Weihua Zhang

**Affiliations:** 1 Department of Epidemiology and Biostatistics, Imperial College London, London, W2 1PG UK; 2 Department of Cardiology, Ealing Hospital, London North West University Healthcare NHS Trust, Middlesex, UB1 3HW UK; 3 National Heart and Lung Institute, Imperial College London, London, W12 0NN UK; 4 Imperial College Healthcare NHS Trust, London, W12 0HS UK; 5 MRC-PHE Centre for Environment and Health, Imperial College London, London, W2 1PG UK

**Keywords:** COVID-19, Polygenic Risk Score, Black Asian Minority Ethnic, susceptibility, severity, genetic risk

## Abstract

**Background:**

COVID-19 manifests with huge heterogeneity in susceptibility and severity outcomes. UK Black Asian and Minority Ethnic (BAME) groups have demonstrated disproportionate burdens. Some variability remains unexplained, suggesting potential genetic contribution. Polygenic Risk Scores (PRS) can determine genetic predisposition to disease based on Single Nucleotide Polymorphisms (SNPs) within the genome. COVID-19 PRS analyses within non-European samples are extremely limited. We applied a multi-ethnic PRS to a UK-based cohort to understand genetic contribution to COVID-19 variability.

**Methods:**

We constructed two PRS for susceptibility and severity outcomes based on leading risk-variants from the COVID-19 Host Genetics Initiative. Scores were applied to 447,382 participants from the UK-Biobank. Associations with COVID-19 outcomes were assessed using binary logistic regression and discriminative power was validated using incremental area under receiver operating curve (ΔAUC). Variance explained was compared between ethnic groups via incremental pseudo-R^2^ (ΔR^2^).

**Results:**

Compared to those at low genetic risk, those at high risk had a significantly greater risk of severe COVID-19 for White (odds ratio [OR] 1.57, 95% confidence interval [CI] 1.42–1.74), Asian (OR 2.88, 95% CI 1.63–5.09) and Black (OR 1.98, 95% CI 1.11–3.53) ethnic groups. Severity PRS performed best within Asian (ΔAUC 0.9%, ΔR^2^ 0.98%) and Black (ΔAUC 0.6%, ΔR^2^ 0.61%) cohorts. For susceptibility, higher genetic risk was significantly associated with COVID-19 infection risk for the White cohort (OR 1.31, 95% CI 1.26–1.36), but not for Black or Asian groups.

**Conclusions:**

Significant associations between PRS and COVID-19 outcomes were elicited, establishing a genetic basis for variability in COVID-19. PRS showed utility in identifying high-risk individuals. The multi-ethnic approach allowed applicability of PRS to diverse populations, with the severity model performing well within Black and Asian cohorts. Further studies with larger sample sizes of non-White samples are required to increase statistical power and better assess impacts within BAME populations.

**Supplementary Information:**

The online version contains supplementary material available at 10.1186/s12920-023-01584-x.

## Background

Coronavirus disease 2019 (COVID-19) is a highly infectious disease caused by severe acute respiratory syndrome coronavirus 2 (SARS-CoV-2). The virus has spread globally since its emergence in Wuhan, China in late 2019, reaching the United Kingdom (UK) in January 2020 [1]. The UK represents one of the most severely impacted countries in Europe. As of May 2022, the pandemic has amassed over 22.3 million cases in the UK alone; associated morbidity and mortality have inflicted health-related burdens of over 177,000 deaths and 860,000 hospital admissions [2]. Resulting strains on healthcare systems and social, economic, and political spheres have been profound [3]. As the UK emerges from the pandemic, a large body of public health research remains focused upon understanding COVID-19 and protecting the population from its future impacts.

An important and unusual manifestation of COVID-19 is the observed heterogeneity in outcomes. Severity of phenotypic presentation ranges from asymptomatic to acute respiratory distress and death [4]. Individual differences in susceptibility to COVID-19, defined as the probability of developing COVID-19 after SARS-CoV-2 exposure, are also widely established [4, 5]. A comprehensive understanding of factors underpinning these patterns can inform population risk stratification and implementation of mitigatory measures to protect those most vulnerable [6]. Alongside external factors such as viral characteristics and efficacy of healthcare and governmental responses, evidence has proven the role of host-associated factors such as older age, male sex, lower socioeconomic status and presence of common comorbidities including hypertension and diabetes in driving COVID-19 susceptibility and severity outcomes [7, 8]. However, it remains that the huge variance cannot solely be explained by these risk factors.

Furthermore, Black, Asian and minority ethnic (BAME) groups within the UK have suffered higher age-standardised diagnosis rates, hospitalisations, and as much as two-fold increases in mortality compared to counterparts of White ethnicity during the pandemic [9]. There has been widespread criticism and demand for policymakers to take further action in protecting minorities bearing high risk burdens [10]. Association of BAME groups with various factors including greater deprivation index, lower vaccine uptake, high-risk frontline occupation, larger multigenerational households, and higher comorbidity burdens have helped in explaining ethnic discrepancies [11, 12]. However, it remains unclear whether genetic differences could also play a role in the increased risk of susceptibility and severity observed in BAME individuals [13]. In order to fully address the long-standing health disparities exacerbated by the pandemic, it is imperative to achieve a more robust understanding of contributory causes [12].

Alongside ethnic differences, family clustering of severe cases and presentation of severe disease among young, healthy patients further supports the possibility of a complex genetic predisposition to adverse COVID-19 outcomes [14]. As susceptibility and severity of infectious and immune-mediated disease can be strongly heritable, investigating host genetic determinants that may impact COVID-19 presentation is vital [15, 16]. Many recent genome-wide association studies (GWAS) are centred around identification of single nucleotide polymorphisms (SNPs) that influence complex disease presentation and pathology [17]. SNPs represent a single point mutation in which one DNA nucleotide is substituted for another; though the majority of variants are silent, others can modulate downstream gene expression and signalling, producing potential pathological impacts [18]. The added contributions of many SNPs with small effects can drive disease development and progression [17]. Individual genetic differences could therefore provide further explanations regarding variability in the context of COVID-19.

The COVID-19 Host Genetics Initiative (HGI) is leading the global effort to meta-analyse results from many COVID-19 GWAS in order to identify important SNPs associated with infection, hospitalisation and death [19]. By comparing variant expression across millions of COVID-19 patients and healthy population controls, results have implicated different sets of variants in influencing COVID-19 susceptibility and severity respectively. Expression of certain SNPs confers increased risk whilst others produce protective effects [15]. COVID-19 SNPs are associated with processes such as innate antiviral defence signalling, mediation of inflammatory organ damage and cell-receptor upregulation; modulation of such pathways can alter infection and subsequent disease phenotype [15, 20]. In addition, studies have proven that variation in COVID-19 SNPs exists between ethnic groups; risk variants associated with the 3p21.31 locus, which confers a greater risk of respiratory failure from COVID-19, are carried disproportionately by individuals of South Asian descent, potentially correlating with high levels of severe COVID-19 within this group [21]. Other variant differences conferring additional risk have been reported within other ethnic groups, including those of African descent [22]. As such, genetic differences could contribute to ethnic disparities in outcomes.

SNPs typically produce modest disease associations when considered individually. However, summation of cumulative SNP effects can represent a greater proportion of polygenic disease risk, and better explain population variance in incidence and severity [23]. A Polygenic Risk Score (PRS) can be utilised to aggregate effects of multiple SNPs into a singular score for pragmatic application to individuals within a population [24]. Broadly, PRS is calculated by summing the number of risk SNPs carried by an individual, weighted by the estimated effect of each variant. SNPs and effect sizes associated with the disease of interest are extracted from a training base dataset, typically GWAS summary statistics, for incorporation into the PRS model; selected variants are then applied to individuals within a distinct target cohort.^(25)^ PRS can therefore be a powerful tool for determining an individual’s genetic liability for developing a particular trait or disease [24, 25]. Scores are typically normally distributed within a population, with a higher score indicating a greater genetic risk [26].

PRS has been applied to many common polygenic pathologies including cardiovascular disease, psychiatric conditions, and cancer, with indication of potential population health benefits [27–29]. Addition of PRS to traditional risk factor models enhances ability to effectively identify high-risk individuals. Clinical implementation of PRS could help to facilitate early detection, define life-time risk trajectory, and deliver targeted interventions [30].

The possibility of developing and applying a PRS using COVID-19 variants has been explored by a small number of studies, with clear associations elicited between PRS and severe disease risk. However, PRS models in most instances have been applied to target cohorts consisting only of European ancestry participants [31–33]. This prevents any assessment of applicability to other ethnic groups or contribution to disparities in outcomes. Though one recent study focusing on European ancestry samples did additionally apply a PRS of 6 SNPs to African and South Asian groups, associations found with COVID-19 outcomes were limited, severely restricted by sample size, and largely non-significant [34]. Moreover, no current UK-based analysis has yet developed and evaluated a PRS model for COVID-19 susceptibility, as recent work has focused on severe disease.

This gap is reflective of a severe underrepresentation of diverse populations within PRS analyses, with very few studies applying PRS models to non-European target cohorts [35]. PRS performs best when base and target samples are ancestry-matched [25]; as there is a severe deficiency of GWAS data from non-European samples, target cohorts of European-descent are typically selected [35]. Generalisability of European-derived PRS to non-European samples is limited due to genetic differences, leading to historically poor performance of PRS within diverse ancestries [36]. Such approaches are damaging and non-inclusive, with potential to exacerbate existing health disparities and prevent advances in genomics and personalised medicine from reaching minority ethnic groups [37]. Recent evidence supports the use of ‘multi-ethnic’ PRS models in order to enhance applicability and predictive accuracy of PRS within diverse populations [35, 38, 39]. This methodology involves utilising training GWAS data that combines samples from multiple population sources across different ancestries, thereby producing significant improvements in PRS performance across diverse ethnicities [35].

Our study aimed to employ this multi-ethnic approach in order to better understand the role of genetics in contributing to COVID-19 susceptibility and severity outcomes across ethnic groups. To facilitate this, two separate PRS were developed: one for susceptibility and another for severity, and applied to a UK-based target cohort. Associations with COVID-19 were tested, and relative predictive performance and explained variance were compared across ethnic groups.

## Methods

In accordance with existing recommendations [25], SNPs and associated beta-value effect sizes showing significant associations with COVID-19 susceptibility and severity outcomes were extracted from the COVID-19 Host Genetics Initiative (HGI) meta-analysis and applied to individual genotype data within the UK Biobank. PRSs for each participant were calculated by computing the sum of risk SNPs present weighted by effect size. Associations, variance explained, and discriminative power were subsequently assessed through statistical analysis. An overview of the employed protocol is outlined in Supplementary Material I.

### Target data sample

The UK Biobank (UKBB) is a prospective population-based cohort study, consisting of over 500,000 participants aged between 40–69 years. Extensive socio-demographic, lifestyle and health-related phenotypic data was collected via surveys and anthropometric measurements across 22 UK assessment centres between 2006 and 2010 [40]. Blood samples were collected, then extracted DNA was directly genotyped using the Affymetrix UK BiLEVE Axiom Array and UK Biobank Axiom Array. Imputation of genotypes was subsequently conducted using the Haplotype Reference Consortium and UK10K haplotype resource, providing a total of around 96 million testable variants [41]. The imputed genotype dataset was utilised for our PRS analysis.

All participants provided informed consent at recruitment for long-term anonymised data storage and health-record access [40]. UKBB holds ethical approval granted by the North West Multi-centre Research Ethics Committee [42] (https://www.ukbiobank.ac.uk/ethics).

Participants who withdrew from the study, were lost to follow-up or who died before January 31^st^ 2020, the beginning of the UK COVID-19 pandemic, were excluded. Those with poor quality or missing genotype data were also removed. Remaining participants who met quality control standards were then stratified by ethnic group to facilitate subgroup analysis.

**Fig. 1:**
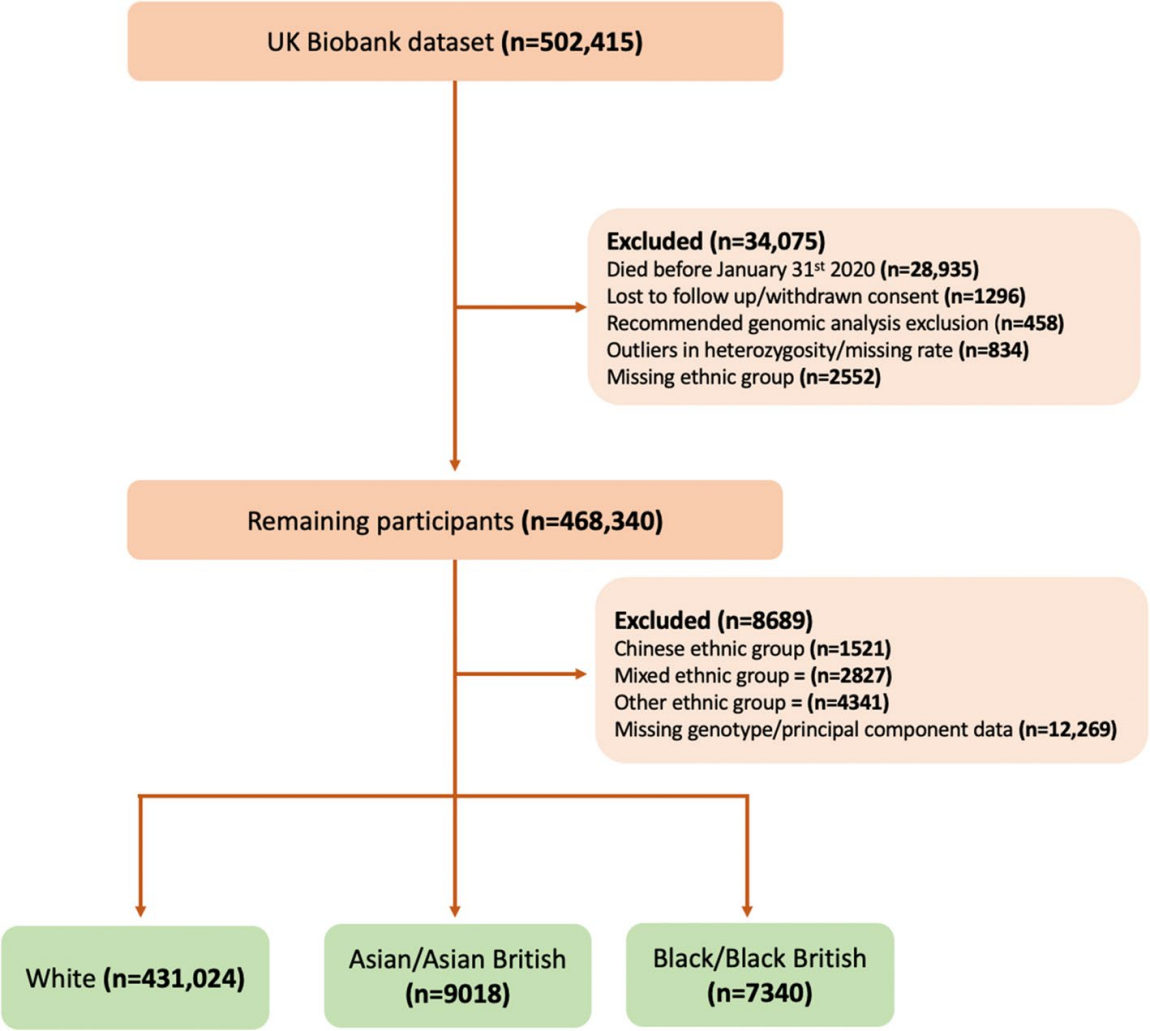
Shows a flow diagram of the included and excluded samples from the UK Biobank cohort. n indicates numbers of UK Biobank participants

Ethnicity was self-reported at enrolment; all UKBB participants identified as one of six ethnic groups before specifying a more specific ethnic background [43]. The groups and corresponding backgrounds utilised for PRS analysis were White (British, Irish and any other White background), Asian/Asian British (Indian, Pakistani, Bangladeshi and any other Asian background) and Black/Black British (Caribbean, African and any other Black background). Mixed, Chinese, and Other ethnic groups were excluded due to a low sample size and associated COVID-19 caseload (Fig. 1).

### Selection of single nucleotide polymorphisms

The leading variants published from Release 6 (June 2021) of the COVID-19 Host Genetics Initiative (HGI) meta-analysis were utilised for our PRS model, consisting of updates to multi-ethnic meta-analysis results [44]. HGI meta-analysed GWAS summary statistics from 61 studies across 24 countries, with an effective sample size over 2 million COVID-19 patients and controls [19]. Variants were stratified by their apparent effects on susceptibility or severity into datasets C2 (infected vs population) and B2 (hospitalised covid vs population). HGI published variants which produced the most significant associations; 9 SNPs associated with susceptibility and 17 with severity were employed for incorporation into the PRS. Selected SNPs and associated beta values are detailed within Supplementary Material II.

### Establishing COVID-19 outcomes

The UKBB data utilised for this study included COVID-19 tests and deaths from the start of the pandemic until November 2021. Participants with any one or more of a) positive COVID-19 test result, b) hospitalisation with COVID-19, and/or c) death from COVID-19 were coded as having a positive susceptibility outcome. Those with one or more of b) hospitalisation with COVID-19 and/or c) death from COVID-19 were coded as having a positive severity outcome. Participants lacking associated COVID-19 data were assumed to be negative for both outcomes.

Outcomes for Biobank participants were ascertained using available dynamically linked electronic health record data. A positive test result was determined using real-time PCR COVID-19 diagnoses from Public Health England or Public Health Scotland, and inpatient diagnoses from hospital data. Hospitalisation with COVID-19 was defined by documented ICU admissions obtained from hospital episode statistics. Death from COVID-19 was defined by death register information showing a death up to 14 days after a positive SARS-CoV-2 test result, or where the underlying cause of death was stated as COVID-19 (ICD-10 codes U0.71 and U0.72) [45].

### Quality control and PRS calculation

All utilised HGI variants had an imputation INFO score of > 0.6 and a minor allele frequency > 0.1% [19], and as such met recommended quality control standards [25]. These parameters were also applied to the target genomic UKBB data, as well as filtering for a Hardy–Weinberg equilibrium of 1 × 10^–6^ and removing SNPs and individuals with a high fraction of genotype missingness.

PLINK 1.90 software [46] was utilised for all genotype extraction and PRS calculation. All target genotype data was converted to PLINK-executable binary format. Selected risk SNPs (Supplementary Material II) for susceptibility and severity were applied to the target UKBB imputed genotype dataset, stratified by ethnic group. Corresponding beta-values and p-values from COVID HGI summary statistics for included SNPs were inputted in order to calculate a PRS for each individual in the UKBB cohort.

### Selection of covariates

Covariates were selected for incorporation into the regression model as potential confounders based on established influences on susceptibility and severity outcomes [47]. Age, sex, alcohol status, smoking status and average total household income were collected via surveys at recruitment for all UKBB participants and subsequently categorised. Body mass index (BMI) in kg/m^2^ was calculated for all individuals from measured height and weight. Townsend deprivation index (TDI) represents a composite measure of socioeconomic status; the score for each participant was derived from collected data regarding home ownership, vehicle ownership, unemployment, and household overcrowding [48]. Comorbidity was ascertained utilising ICD-10 codes from hospital records; coronary heart disease (CHD, ICD-10 I121-I123) type 2 diabetes mellitus (T2DM, ICD-10 E11), hypertension (ICD-10 I10-I15) and respiratory disease (Chronic Obstructive Pulmonary Disease (COPD) and/or asthma diagnosis, ICD-10 J41-J45) were added as confounders. Any previous diagnoses of these conditions from available linked hospital statistics up to 31^st^ August 2021 were counted as a positive outcome.

Univariate logistic regression of each covariate separately against COVID-19 infection and severity outcomes was undertaken; all associations were significant, and all covariates were incorporated into the final model as confounders. No collinearity was found between included variables. The first 10 principal genetic components (PCs) of each participant were also included as covariates to adjust for population genetic structures and avoid bias, as per current recommendations [25, 26].

### Statistical analysis and association testing

Baseline characteristics for participants within each ethnic group were calculated as numbers of cases, percentages and means with standard deviations.

Once PRS was calculated, each ethnic group was separately stratified into quintiles for susceptibility and severity PRS, then categorised into low genetic risk (quintile 1, bottom 20% of cohort), intermediate risk (quintiles 2–4, middle 60%) and high risk (quintile 5, top 20%) for each outcome. Binomial logistic regression of PRS risk categories against COVID-19 susceptibility and severity outcomes was then conducted using SPSS v.27, fully adjusted for confounders. For each regression, odds ratios (ORs) and Wald’s test p-values were described. Nagelkerke pseudo-R^2^ was reported for regression models incorporating PRS and covariates, and for covariates alone. The incremental pseudo-R^2^ (ΔR^2^) was calculated as the difference between the two models, reported as the proportion of variance explained by PRS alone.

Discriminative power of models in identifying high-risk individuals was then assessed using receiver operating curve (ROC) analysis. Area under the receiver operating curve (AUC) was calculated for full models (consisting of covariates and PRS) and base models (covariates only). Increment in AUC (ΔAUC) was reported based on the difference between the two models, reported as the discriminative or predictive power conferred by PRS.

One-way ANOVA tests were conducted to assess differences between mean susceptibility and severity PRS between ethnic groups. As PRS demonstrated a normal distribution (Supplementary Material III) and showed non-homogeneity in variance between ethnic groups, a post-hoc Games-Howell multiple comparisons test was selected for subsequent pairwise analysis.

All analyses are reported according to existing guidance [49].

## Results

### Descriptive characteristics of study participants

Table 1 shows participant demographics within each ethnic group. 96.4% of the overall cohort were of White ethnicity, 2.0% Asian and 1.6% Black. Incidence of COVID-19 infection was 4.6% higher within the Asian cohort and 3.1% higher within the Black cohort as compared to the White cohort. Similarly, severe COVID-19 incidence was 0.9% and 1.3% greater in Asian and Black groups respectively.

**Table 1:**
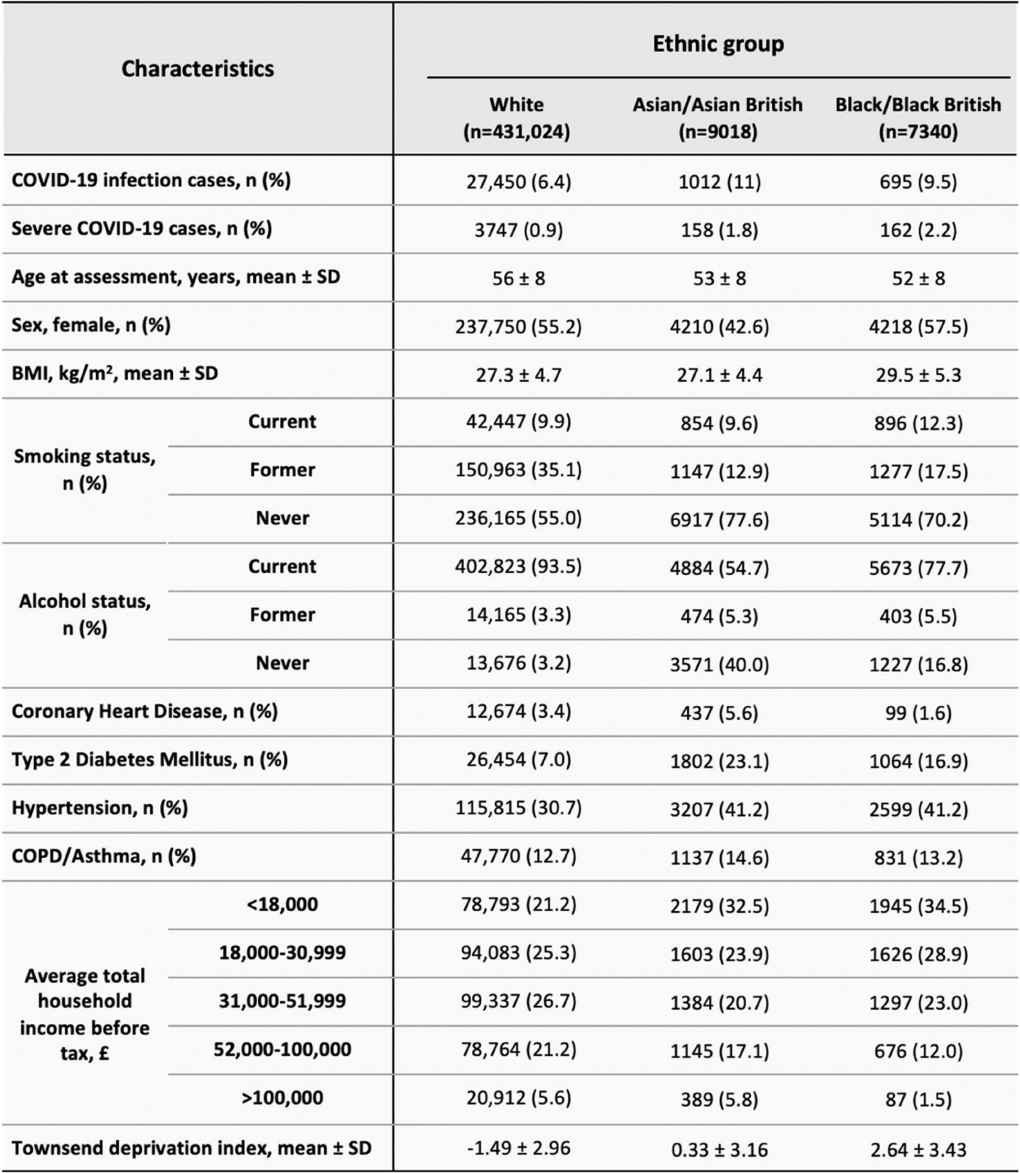
Shows the demographic characteristics of UK Biobank participants stratified by ethnic group. n (%) refers to the number of cases showing the characteristic, followed by the number expressed as a percentage of the total number of participants within the ethnic group

*Abbreviations: *SD Standard deviation of mean, *BMI* Body mass index, *COPD* Chronic obstructive pulmonary disease

Ages were comparable across all ethnicities. The Asian group comprised a comparatively lower proportion of female participants (42.6%). The Black cohort represented the highest mean BMI (29.5) and the greatest proportion of current smokers (12.3%), while the White group had the greatest proportion of current alcohol consumers (93.5%). Asian participants showed the greatest incidence of CHD, T2DM and COPD/Asthma. The Black group was associated with the lowest household income and greatest deprivation index.

### Testing associations between PRS and incident COVID-19 outcomes

After within-ethnicity stratification of participants into PRS genetic risk categories was completed, binomial logistic regression was performed.

Table 2A shows the results from the regression of the fully adjusted PRS model against incident severe COVID-19. A significant association between PRS and severe COVID-19 incidence was found for all ethnicities; overall p-values were 1.35 × 10^–19^, 1.33 × 10^–3^ and 2.36 × 10^–2^ for the White, Asian, and Black cohorts respectively (*p* < 0.05). The White group demonstrated the greatest overall significance (*p* < 0.0001). Odds ratios (ORs) relative to the low genetic risk category were greatest within the Asian cohort (1.97-fold risk for intermediate, 2.88 for high) and Black cohorts (1.99 for intermediate, 1.98 for high) as compared to the White cohort (1.17 for intermediate, 1.57 for high). Associated 95% confidence intervals were universally largest within the Asian group, followed by Black and White cohorts. Pseudo-R^2^ for full models including PRS were 10.8%, 17.3% and 12.7% for White, Asian, and Black cohorts respectively. The Asian cohort showed the greatest ΔR^2^ between base and full models, with 0.98% of total variance in severity explained by PRS alone. PRS explained 0.61% and 0.20% of variance in Black and White cohorts respectively.

**Table 2:**
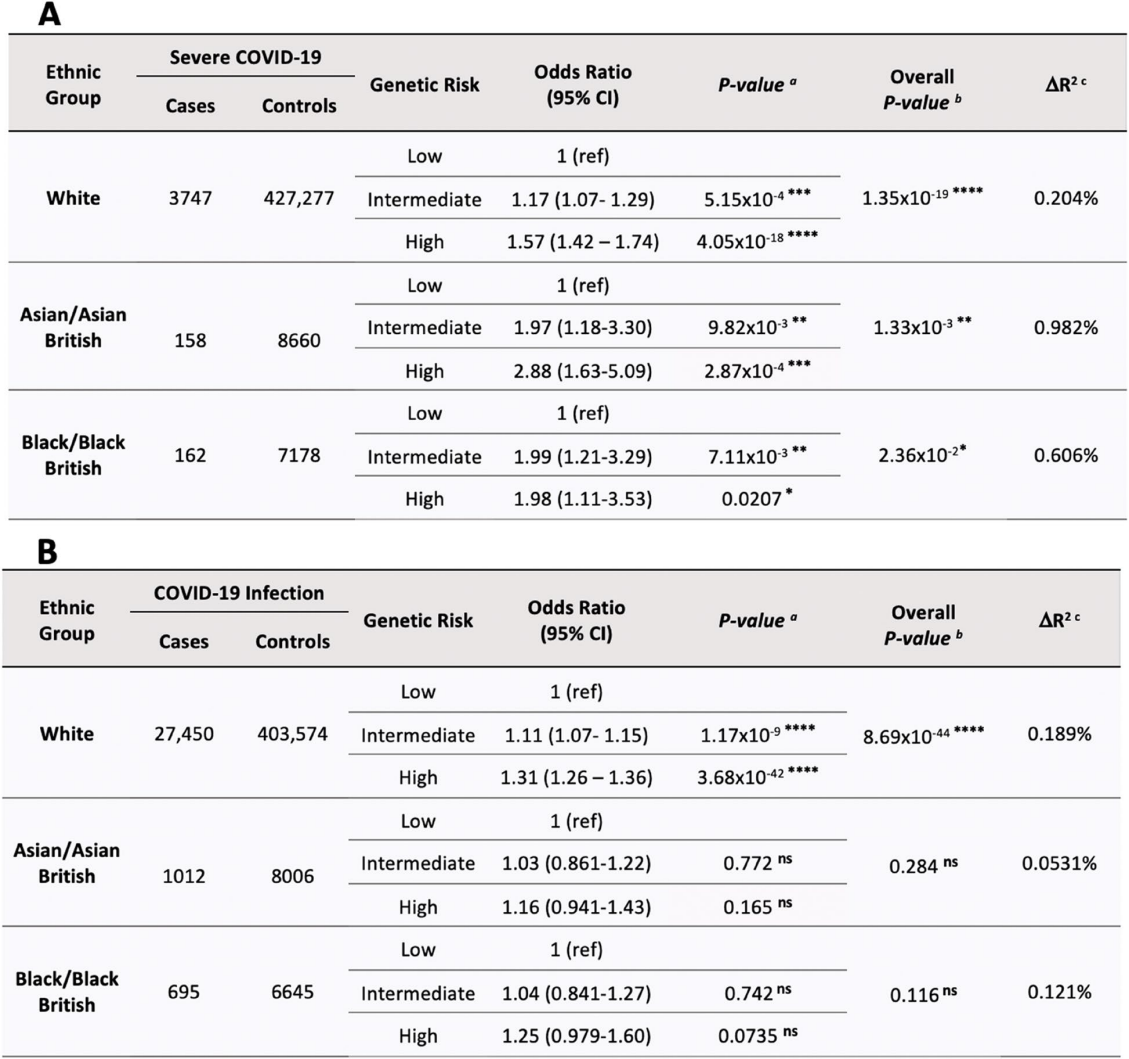
Shows the results of binomial logistic regression for PRS, fully adjusted for confounders, against A) Severe COVID-19 and B) COVID-19 infection

*Abbreviations: CI* Confidence intervals

^a^ *p*-value relative to the low genetic risk category. ^b^ Overall p-value for association of PRS as an independent variable with COVID-19 outcome. ^c^ Incremental difference in Nagelkerke pseudo-R^2^ between full model (covariates + PRS) and base model (covariates alone)

* *p* < 0.05, ** *p* < 0.01, ***p < 0.001, ****p < 0.0001, ^ns^ no significance

Table 2B shows results from the regression of susceptibility PRS category against COVID-19 infection cases, including the same confounders. A highly significant overall association between PRS and COVID-19 infection was shown for the White ethnic group (*p* = 8.69 × 10^–44^, < 0.0001). Associations for Asian and Black cohorts were non-significant (*p* > 0.05). Odds of COVID-19 infection relative to the low-risk category were increased for the White cohort (1.1-fold risk for intermediate, 1.31 for high). All ORs and ΔR^2^ were less than those demonstrated with severity PRS. Full model pseudo-R^2^ values including PRS were 3.0%, 4.9% and 3.1%, demonstrating incremental increases of 0.19%, 0.053% and 0.12% from the base model for White, Asian, and Black cohorts respectively.

### Receiver Operating Curve (ROC) analysis

All analyses showed small improvements in area under the receiving operating curve (AUC) with addition of PRS to the base model containing covariates only, illustrating an improvement in ability to predict COVID-19 susceptibility and severity across all ethnicities. Across all models, asymptotic *p*-value was < 0.0001, indicating a statistically significant ability to predict risk.

**Fig. 2:**
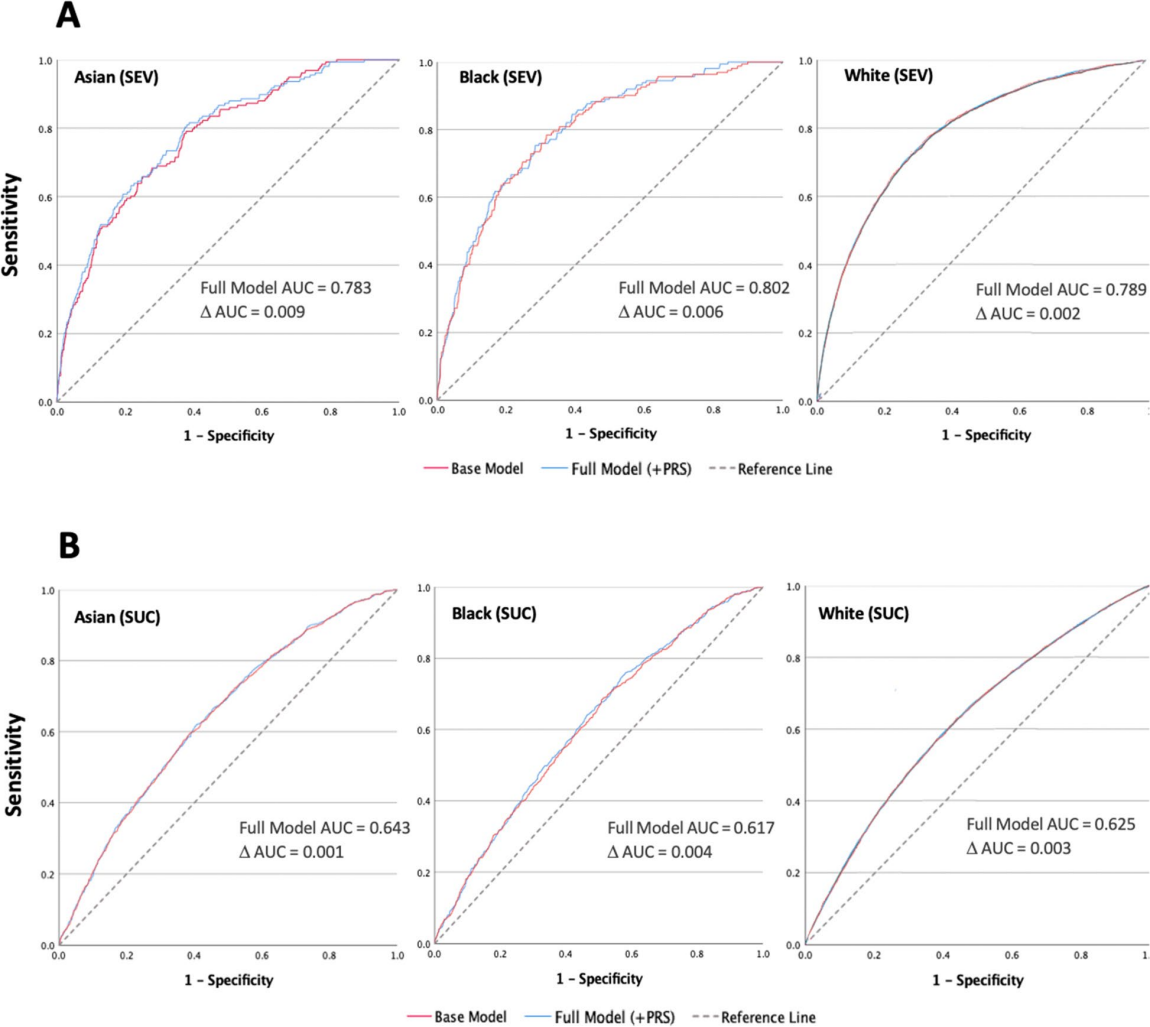
Shows receiver operating characteristic (ROC) curves for base models (covariates only) and full models (covariates + PRS) for A) severity and B) susceptibility PRS across all ethnic groups. Area under the curve (AUC) for the full model, and incremental increase in AUC (ΔAUC) from base model is reported for each curve. An AUC of 0.5 indicates the model produces no utility in predicting risk. Abbreviations: SEV = COVID-19 severity, SUC = COVID-19 susceptibility

Figure 2A shows that for severity, PRS produced a 0.9%, 0.6% and 0.2% improvement in AUC for the Asian, Black and White cohorts respectively. This demonstrates enhanced discriminative power of PRS within non-White cohorts.

For susceptibility, the greatest incremental AUC was demonstrated by the Black cohort, followed by White and Asian cohorts (Fig. 2B). All observed AUCs were smaller than those for severity.

### Evaluating relationship between PRS and COVID-19 risk

**Fig. 3:**
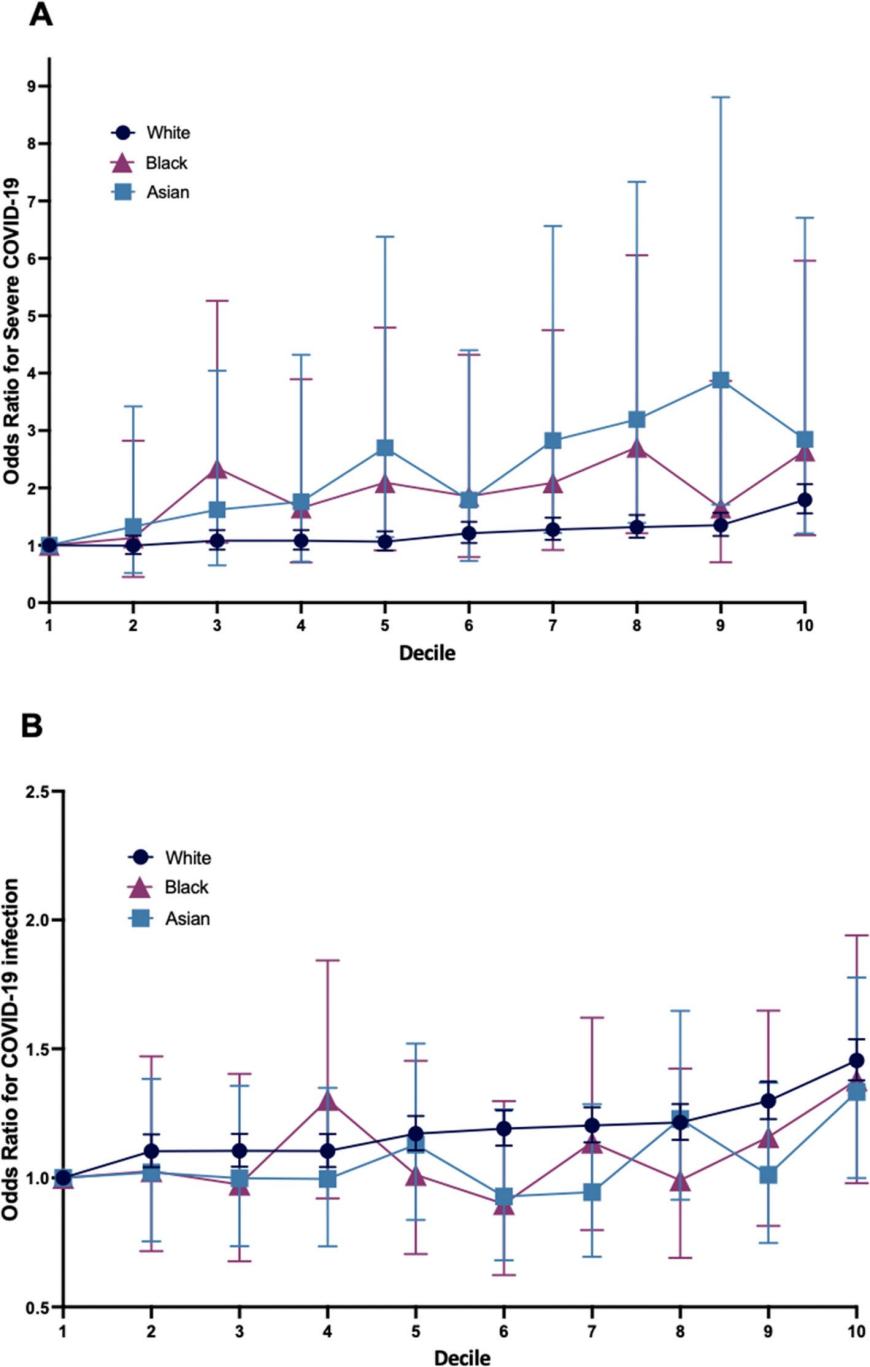
Is a quantile plot of PRS percentiles against odds ratios for A) Severe COVID-19 and B) COVID-19 infection, relative to the first percentile for all ethnicities. Error bars show 95% confidence intervals for odds ratios

In order to evaluate if increases in PRS across smaller strata were associated with observable increases in risk, susceptibility and severity PRS were further stratified into deciles and plotted against ORs relative to the first decile for severe COVID-19 (Fig. 3A) and COVID-19 infection (Fig. 3B) respectively.

For severity, the White cohort showed an increase in risk across deciles with the most substantial increases present at the tail of the distribution. Black and Asian cohorts also demonstrated overall increases in risk with generally higher ORs, though increases across deciles were more sporadic with prominent fluctuations. Notably, OR decreased between ninth and tenth deciles for the Asian cohort. Confidence intervals associated with Black and Asian cohorts were very large.

For susceptibility, only the White group demonstrated an increase in OR across deciles. Similarly, this increase was most pronounced at the highest deciles. Black and Asian cohorts demonstrated no uniform trend, though risk increased in both groups across the highest decile.

### Differences in mean distribution between ethnic groups

**Fig. 4:**
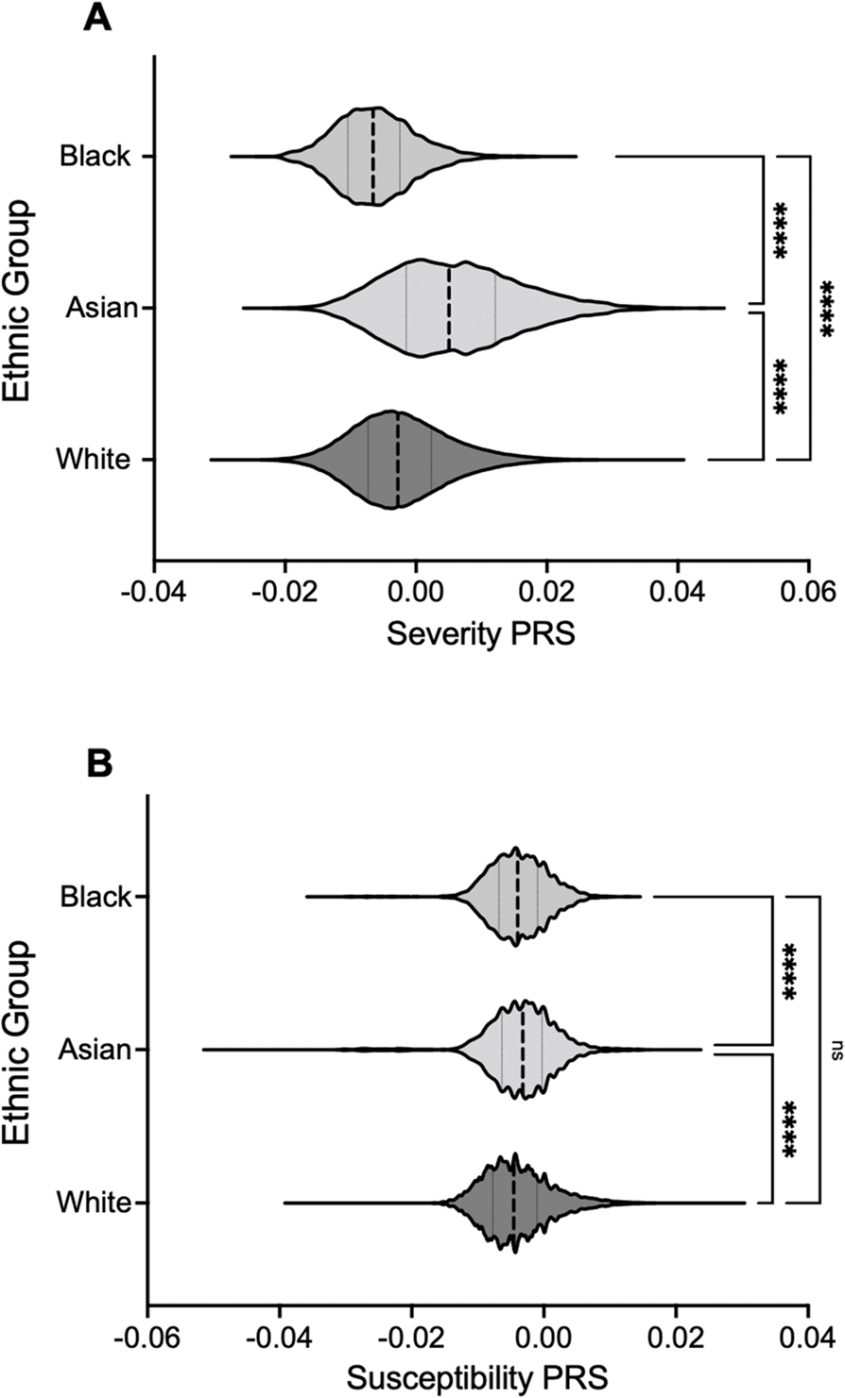
Is a violin plot showing the differences in A) COVID-19 Severity and B) COVID-19 Susceptibility PRS distributions for different ethnicities. Central dashed lines within each violin indicate medians, with the other two lines indicating upper and lower quartiles. **** = *p* < 0.000, ns = no significance for Games-Howell multiple comparisons test

PRS was normally distributed for all ethnicities (Supplementary Material III). Figure 4 shows the difference in PRS distributions across ethnicities, and results of the one-way ANOVA and post-hoc Games-Howell test for significant differences between means.

For severity (Fig. 4A) the Asian ethnic group showed the highest mean PRS (5.78 × 10^–3^), followed by White (2.14 × 10^–3^) and Black (-6.22 × 10^–3^); differences between all groups were significant (*p* < 0.0001).

Figure 4B shows that Asian participants again demonstrated a significantly greater mean susceptibility PRS than other groups (-3.43 × 10^–3^, *p* < 0.001), followed by Black (-3.92 × 10^–3^), then White (-4.02 × 10^–3^). Differences between the latter groups were non-significant (*p* = 0.177).

## Discussion

Within this study, two separate PRSs were calculated based on leading variants associated with COVID-19 susceptibility and severity respectively. Scores were applied to a UK-based target cohort of 447,382 participants across White, Asian/Asian British, and Black/Black British ethnic groups. Meaningful associations were elicited between PRS and corresponding COVID-19 outcomes. Discriminative performance, variance explained, and mean distributions were compared between ethnic groups.

A significant association between severity PRS and incident severe COVID-19 was found across all ethnicities, independent of other confounders. The highest PRS risk categories generally showed highest adjusted odds ratios, implying a direct relationship between PRS and severe disease risk; this establishes a genetic basis for differences in severity outcomes between individuals. These findings align with previous analyses conducted in European-ancestry target cohorts that elicited similar relationships between PRS and severe COVID-19 [31, 32].

Severity PRS performed well across all ethnicities, and exhibited a greater predictive power and variance explained within Asian and Black cohorts. This represents a very promising finding, especially when considering historically poor performance of PRS within diverse populations [35]. Our PRS predicted risk more effectively in non-White cohorts than typical models trained using European-only GWAS samples, illustrating the benefit of the multi-ethnic approach employed. This further validates findings that utilising multi-ancestral GWAS training datasets can allow predictive accuracy within wider ethnic groups [50]. As such, we recommend wider employment of multi-ethnic PRS models to facilitate a more inclusive approach and rectification of current ethnic imbalances in PRS analyses.

Odds ratios and variance explained for susceptibility PRS were universally smaller than for severity across all ethnicities. This may suggest that genetic predisposition contributes less to COVID-19 susceptibility risk as compared to severity, supporting hypotheses that differences in susceptibility outcomes are driven more by factors associated with SARS-CoV-2 exposure, such as occupation and household overcrowding, as opposed to other biological factors [51]. Furthermore, whilst susceptibility PRS and COVID-19 infection were highly associated within the White cohort, non-significant associations and a smaller predictive power were produced within the Black and Asian cohorts. Though this could imply that PRS cannot explain COVID-19 infection burdens in non-White groups, it is more likely that this finding is attributable to the considerably smaller numbers of Asian and Black individuals within the UK Biobank; limited sample size can result in failure to detect associations between PRS and the associated trait [24]. Further research utilising larger sample sizes for non-White cohorts is required to establish more firm conclusions.

As highlighted in Fig. 3A and B, fluctuations in ORs across strata were very large for Black and Asian cohorts as compared to the White cohort for both susceptibility and severity. This inconsistency may be attributable to the markedly smaller sample sizes of these groups, further evidenced by the large confidence intervals associated with the ORs. It remains that with the current model, PRS cannot be reliably implemented for risk stratification in non-White ethnic groups and may produce damaging consequences; for instance, individuals in the Black cohort within the ninth PRS decile for severity produce an odds ratio as low as that of the second decile, illustrating an inability to accurately stratify individuals. Furthermore, this sporadic relationship influenced the initial stratification into risk categories for regression and subsequent odds ratios produced. Those in the eighth severity decile of the Black cohort produced a greater odds ratio for severe COVID-19 than the ninth and tenth deciles but were classified as intermediate risk, with the ninth and tenth deciles classified as high genetic risk. This discrepancy may help to explain the similar odds ratios observed within the intermediate (1.99, CI 1.21–3.29) and high risk (1.98, CI 1.11–3.53) categories for severity PRS regression. As such, further analysis utilising more robust and larger genetic data samples from non-White cohorts is necessary before it may be reliably and feasibly implemented for risk stratification within these groups.

The results do, however, indicate potential utility for the PRS model in risk stratification of the White cohort for both susceptibility and severity, as indicated by the consistent increase across strata seen within this group in Fig. 3A and B. This could assist in protection of those with the greatest genetic vulnerability in potential future outbreaks; targeted public health interventions such as shielding, closer monitoring, protection from high-risk frontline work and vaccination prioritisation may help to mitigate associated risk. Hospital-based applications might facilitate screening of COVID-19 patients and early detection of severe disease [30]. Furthermore, informing patients of an increased polygenetic risk has some evidence of positive behavioural impact [52], with potential to decrease risk-taking behaviours and therefore promote better outcomes.

However, important societal and ethical concerns pertaining to PRS implementation must be considered; prescribing high-risk individuals to continue shielding or abstain from work longer than others may reinforce detrimental impacts on financial security, mental wellbeing and social functioning that outweigh the conferred risk [53]. Additionally, potential discriminatory impacts of genetic risk stratification could include preferential employment of low-risk individuals and increased insurance premiums for those at higher genetic risk [53, 54]. No formal legislation regarding genetic discrimination currently exists in the UK [55]; wider issues must be addressed before clinical implementation of PRS can be considered to avoid marginalisation of those most vulnerable.

Findings also demonstrated significant differences in mean PRS between ethnic groups for both susceptibility and severity. Similar differences have been produced by other studies [55, 56]. This has been attributed to differences in allele frequencies and linkage disequilibrium patterns between ancestries; as variants exist at differing levels within each population, differences in absolute PRS values are produced [57, 58]. Such discrepancies were also present within our analysis, as differences in mean allele frequency for the SNPs utilised were present between ancestry groups within the UKBB cohort (Supplementary Material IV). This suggests that absolute PRS values are not directly transferable between ethnicities, as a score considered high-risk in one group may fall within the lower-risk distribution of a different group. It can be concluded that utility of our PRS is restricted to risk stratification within ethnic groups and must be interpreted relative to population-specific distributions; it cannot be applied across all populations in tandem. Accurately contextualising individual PRSs to correct ancestries for interpretation poses a logistical challenge to clinical implementation [30].

Though meaningful associations were produced, variance in outcomes explained by PRS was under 1% across all analyses. Whilst addition of PRS to existing risk factor models was shown to enhance risk prediction and elucidate some ethnic differences, such small proportions show that genetics alone is by no means explanatory of ethnic disparities. The need to avoid the so-called ‘molecularisation’ of race through placing sole focus upon genetic and biological differences between racial groups has been emphasised; [59] it is evident that more investigation of structural and systemic factors that drive disparities are needed in order to fully understand and mitigate the risk experienced by BAME groups within the UK [11].

Whilst severity PRS performed well in Black and Asian cohorts, it is important to consider implications of the more sporadic relationship observed between PRS strata and COVID-19 risk, and the universally large associated confidence intervals exhibited as compared to the White cohort. These findings, as well as the lack of susceptibility association, are likely attributable to the severely limited sizes of non-White samples. The available UK Biobank population comprised only 2% Asian/Asian British and 1% Black/Black British participants (Table 1); such proportions are not representative of the UK population demographics which are estimated at 8% and 3.5% respectively [60]. Limited statistical power within non-White cohorts reduces confidence in associations and conclusions drawn, indicating the need for further research utilising more robust data from UK BAME groups.

Though the COVID-19 HGI Release 6 meta-analysis utilised was multi-ethnic and global in nature, the effective sample size remained heavily European-dominant [19]. Incorporated SNPs may therefore have a reduced applicability to non-European samples, representing a limitation of our study. This is reflective of the severe deficiency of GWAS and PRS analyses within diverse populations [35]. Research forums have emphasised the need to collect samples from underrepresented ancestries [61], and initiatives such as Polygenic Risk Methods in Diverse Populations (PRIMED) consortium have been recently introduced to promote enhanced PRS risk prediction within broader ethnicities [62]. However, it remains that more must be done to rapidly address inequalities and ensure advances are made suitable for all populations.

Another factor limiting applicability of findings included oversimplification of ancestry within our analysis. Broad ethnic groups were utilised due to the small sample sizes available; however, this overlooked important within-group heterogeneity. For instance, Bangladeshi and Black African individuals experienced significantly worse burdens than other backgrounds from wider Asian and Black ethnicities [63]. Aggregation of backgrounds prevents elucidation of such differences and identification of specific populations at the highest risk. More data is required in order to perform within-group stratification and understand genetic contribution to such patterns. Whilst ethnicity serves as an important proxy for differences in genetic ancestry and societal influences that drive tangible health inequalities between groups, it remains a social construct, and ill-defined within genomics [59]. Further study is required regarding applicability of personalised medicine in the context of imperfect categorisations of race and ethnicity, and how we can most effectively group individuals based on true ancestorial patterns.

Further study limitations comprised inclusion of a limited number of SNPs. Incorporation of larger numbers of variants within the PRS confers a greater predictive performance;[26] whilst we only included 24 variants, this number is greater than those utilised within some other published COVID-19 PRS analyses [34] and so our study can still provide a somewhat more comprehensive model. Furthermore, the summary statistics utilised from the COVID-19 HGI meta-analysis included UK Biobank participants; this overlap in base and target populations can lead to overestimations in prediction accuracy of PRS and represents a limitation in the model used. Additionally, data regarding factors such as occupational exposure and multigenerational households were not available; such factors directly influence ethnic disparities [12] and their inclusion as confounders would enhance conclusions drawn. Furthermore, Chinese, Other and Mixed ethnic groups were excluded from this analysis due to severely limited sample sizes, preventing assessment of performance. Further research regarding genetic factors within these groups is required.

## Conclusion

Our study is the first to prioritise analysis and assessment of a COVID-19 PRS within multiple UK ethnic groups. PRS was significantly associated with severe COVID-19, and higher risks in Asian and Black cohorts can help to explain ethnic disparities in outcomes. A significant association between susceptibility PRS and COVID-19 infection was found for the White cohort. Further analysis utilising larger sample sizes from non-White cohorts is needed to enhance statistical power, increase confidence in conclusions and better assess impacts within BAME groups. A multi-ethnic approach was shown to be beneficial in allowing predictive accuracy of PRS within diverse ancestries, and therefore should be more widely employed.

## Supplementary Information

**Additional file 1**: **Supplementary Material I.** shows the methodology employed for PRS analysis. **Supplementary Material II.** Included Variants. **Supplementary Material III.** Distributions of PRS. **Supplementary Material IV.** Mean Allele Frequencies.

## Data Availability

The participant data that support the findings of this study are available from the UK Biobank but restrictions apply to the availability of this data, which were used under licence for the current study and so are not publicly available. Data are however available from the authors upon reasonable request and with the permission of UK Biobank. The genetic summary statistics data analysed during the current study are available from the COVID-19 Host Genetics Initiative repository, via the following link: https://www.covid19hg.org/results/r6/.
