# Supplementary material for: Associations between polygenic risk score and covid-19 susceptibility and severity across ethnic groups: UK Biobank analysis
Source: BMC Med Genomics. 2023 Jun 30;16:150. doi: 10.1186/s12920-023-01584-x (PMC10311902; doi:10.1186/s12920-023-01584-x)
Supplement: Supplementary file 1 — Additional file 1: Supplementary Material I. shows the methodology employed for PRS analysis. Supplementary Material II. Included Variants. Supplementary Material III. Distributions of PRS. Supplementary Material IV. Mean Allele Frequencies. [file 12920_2023_1584_MOESM1_ESM.pdf]

Supplementary Material

Supplementary Material I – Overview of Methodology

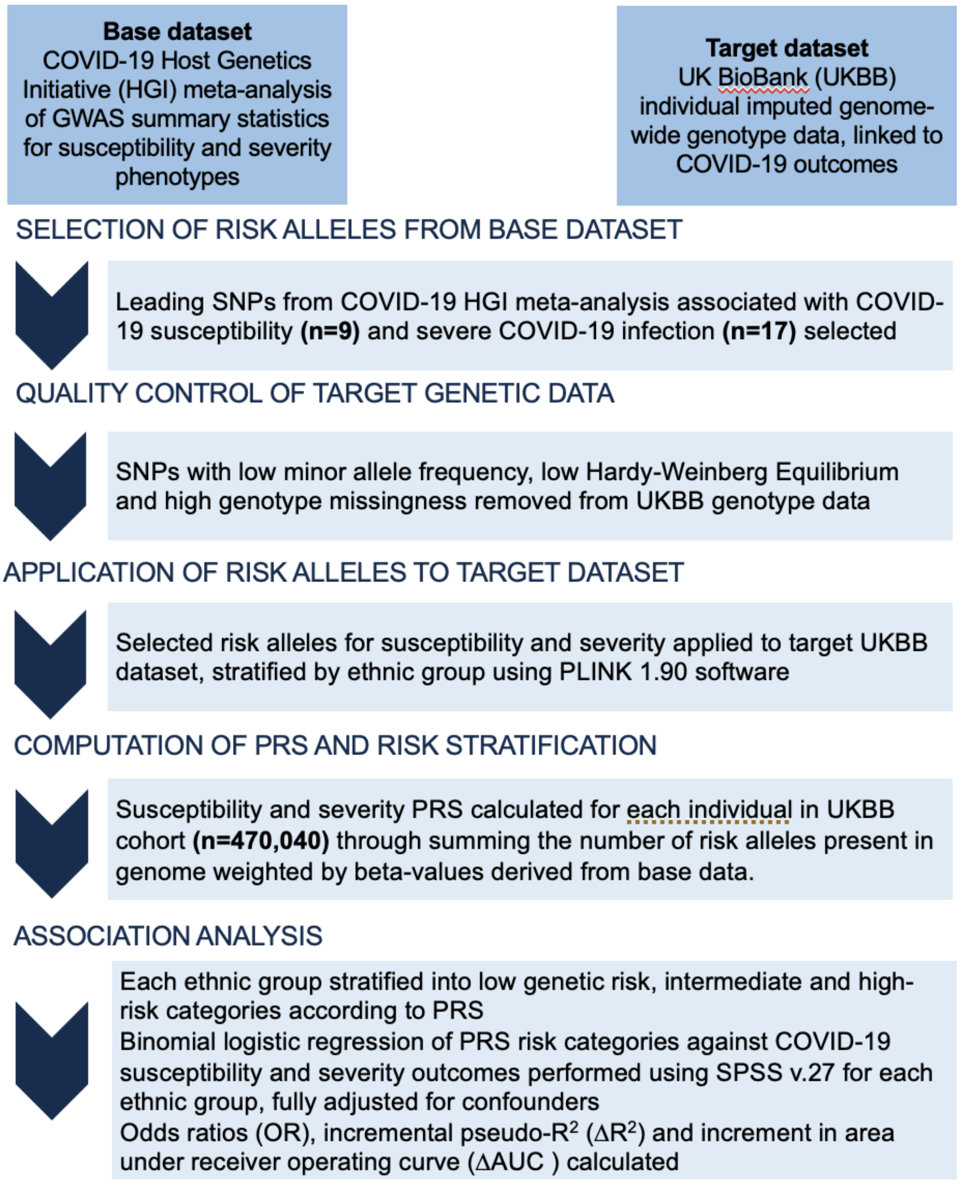

Supplementary Material I *shows the methodology employed for PRS analysis. Abbreviations: PRS = polygenic risk score, SNPs = single nucleotide polymorphisms,*

Supplementary Material II – Included Variants

Supplementary Material II *shows the leading variants from COVID-19 Host Genetics Initiative Release 6 associated with susceptibility and severity outcomes that were incorporated within the polygenic risk score. Abbreviations: SNP = single nucleotide polymorphism*

COVID-19 Susceptibility – infected vs population (C2)						
**SNP**	**Chromosome**	**Associated gene**	**Functional effect**	**Risk allele frequency**	**Meta-p value**	**Beta value**
rs505922	9	ABO	intron	0.6515	$3.41x10^{-82}$	-0.078507
rs73062389	3	SLC6A20	intron	0.04942	$2.53x10^{-74}$	0.16488
rs17412601	3	NXPE3	intron	0.342	$1.68x10^{-24}$	-0.043171
rs190509934	23	ACE2	upstream gene	0.003063	$3.63x10^{-18}$	-0.37606
rs4801778	19	PLEKHA4	intron	0.1741	$8.17x10^{-14}$	-0.039295
rs2109069	19	DPP9	intron	0.308	$1.21x10^{-13}$	0.033082
rs148063273	1	EFNA1	upstream gene	0.008203	$1.93x10^{-9}$	0.13057
rs4767023	12	OAS1	intron	0.663	$2.85Ex10^{-8}$	0.02329
rs2071351	6	HLA-DPB1	intron	0.1909	$4.60x10^{-8}$	0.027248
COVID-19 Severity – hospitalised vs population (B2)						
rs35508621	3	LZTFL1	intron	0.07603	4.81E-100	0.4013
rs13050728	21	IFNAR2	intron	0.658	5.13E-23	-0.10935
rs505922	9	ABO	intron	0.6577	1.71E-22	-0.10323
rs2109069	19	DPP9	intron	0.3075	2.05E-22	0.11214
rs11085727	19	TYK2	intron	0.2734	2.66E-14	0.088793
rs41435745	6	FOXP4-AS1	intron	0.03672	6.85E-13	0.18547
rs10774679	12	OAS3/OAS1	intron	0.6437	6.97E-13	0.077247
rs766826	11	ELF5	intron	0.3345	2.93E-12	-0.082633
rs61667602	17	LINC02210-CRHR1	intron	0.1888	3.77E-12	-0.093206
rs111837807	6	CCHCR1	intron	0.08672	2.33E-11	0.12021
rs77534576	17	RNU6-1313P/TAC4	regulatory region	0.03083	4.29E-11	0.20867
rs1405655	19	NR1H2	intron	0.3341	5.34E-11	0.071207
rs67579710	1	THBS3	intron	0.106	1.76E-10	-0.10634
rs12809318	12	FBRSL1	intron	0.5181	3.22E-09	-0.063638
rs35705950	11	MUC5B	upstream gene	0.1014	6.47E-09	-0.11154
rs721917	10	SFTPD	missense	0.4257	1.69E-08	0.058821
rs117169628	16	SLC22A31	missense	0.137	2.55E-08	0.088157

Supplementary Material III – Distributions of PRS

A

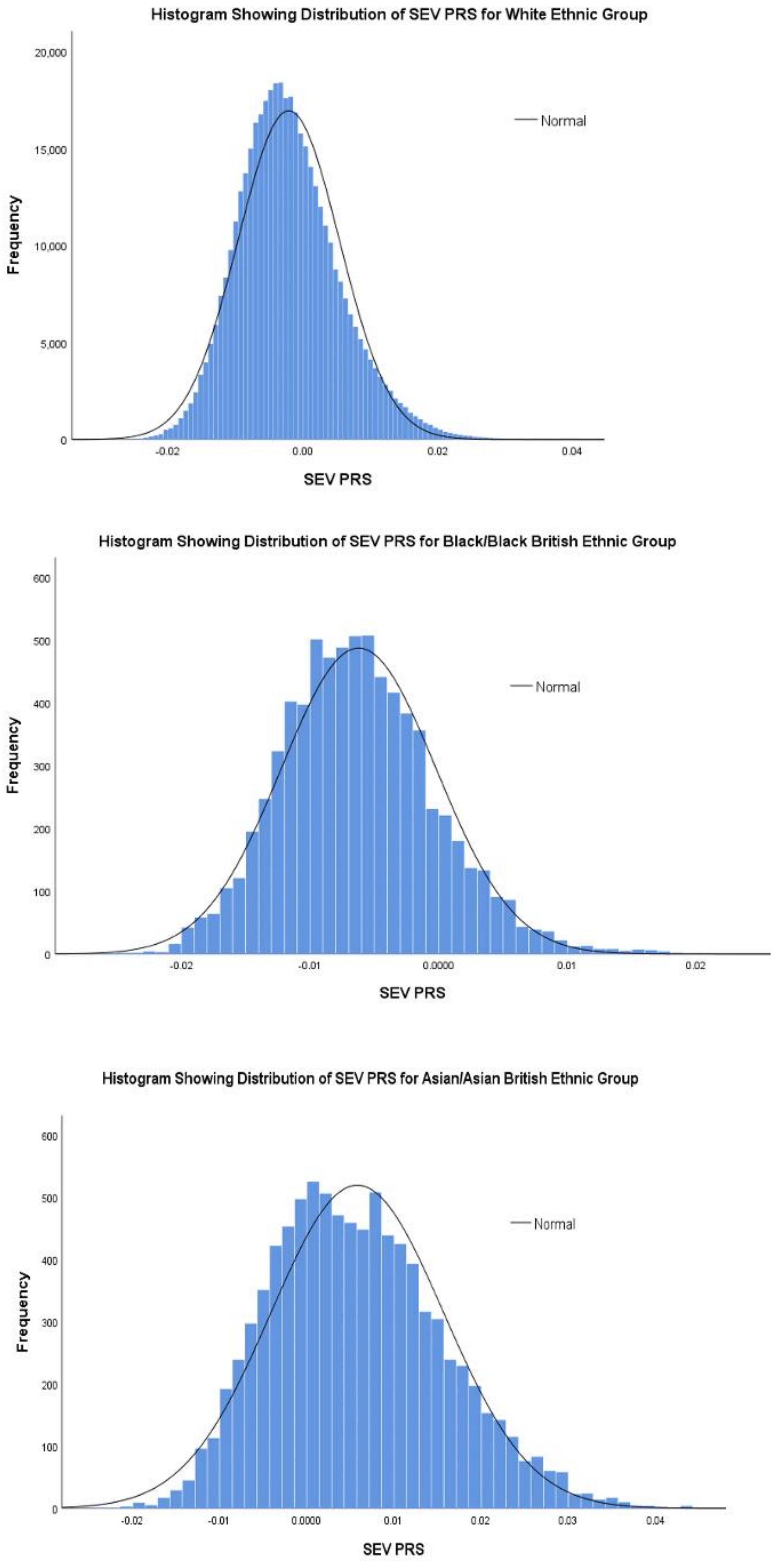

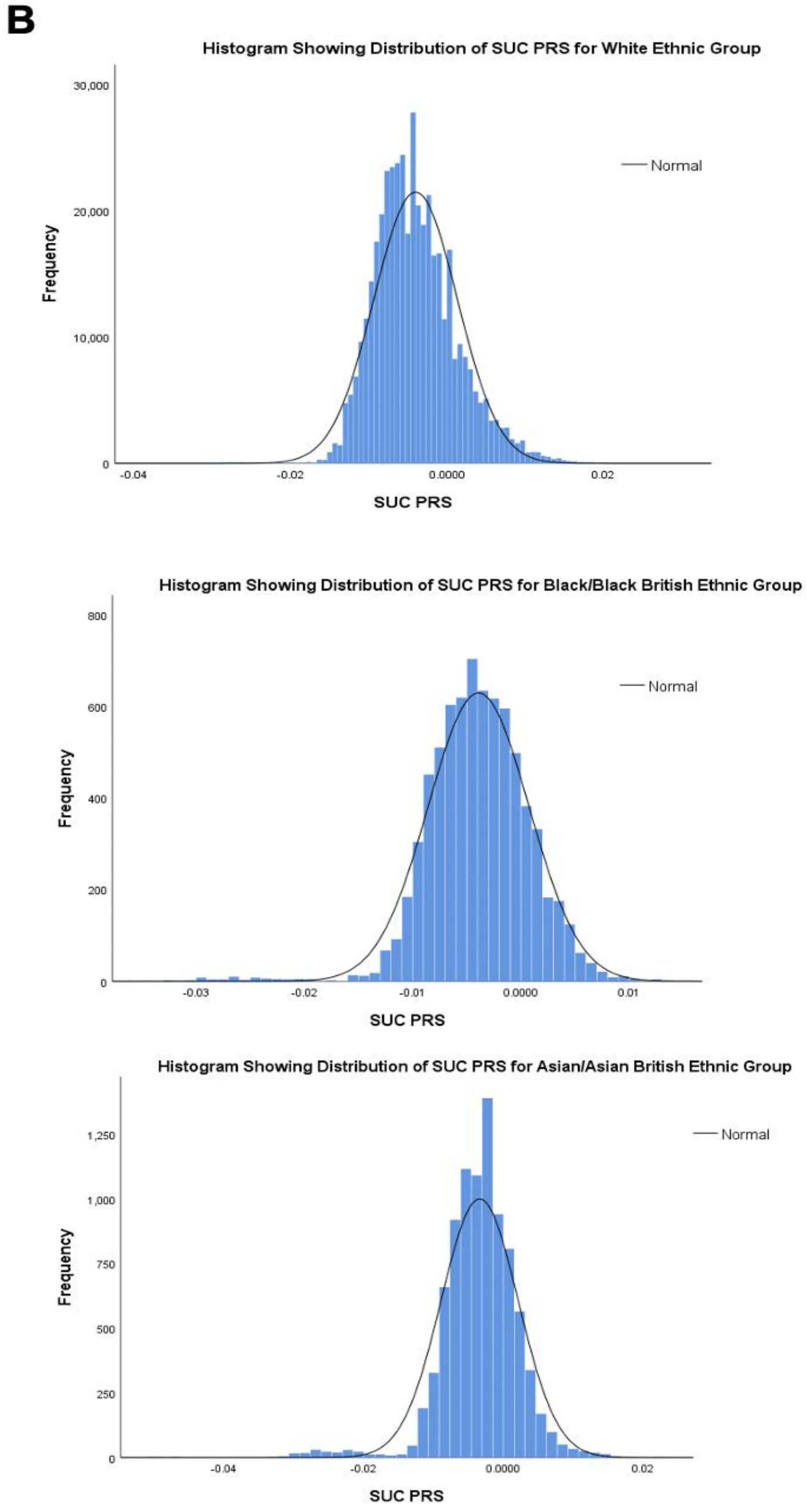

Supplementary Material III *shows the normal distributions of (A) severity and (B) susceptibility polygenic risk scores within each ethnic group. Abbreviations: SEV = severity, SUC = susceptibility*

Supplementary Material IV – Mean Allele Frequencies

Supplementary Material II! *shows the mean allele frequencies of the included SNPs influencing susceptibility and severity for each ethnic group within the UK Biobank population. Abbreviations: SNP = single nucleotide polymorphism*

COVID-19 Susceptibility – infected vs population (C2)			
	**Mean Allele Frequency**		
**SNP**	**Black Cohort**	**White Cohort**	**Asian Cohort**
rs505922	0.3557	0.3176	0.4173
rs73062389	0.006471	0.05977	0.02112
rs17412601	0.2597	0.3522	0.2299
rs190509934	0.006187	0.002088	0.01622
rs4801778	0.1593	0.188	0.1368
rs2109069	0.197	0.322	0.1995
rs148063273	0.0007499	0.008411	0.0004462
rs4767023	0.4639	0.3537	0.3054
rs2071351	0.4384	0.1802	0.3387
COVID-19 Severity – hospitalised vs population (B2)			
rs35508621	0.009336	0.06969	0.2538
rs13050728	0.1994	0.3099	0.474
rs505922	0.3557	0.3176	0.4173
rs2109069	0.197	0.322	0.1995
rs11085727	0.09492	0.2918	0.2719
rs41435745	0.08058	0.01418	0.1113
rs10774679	0.3943	0.3701	0.3063
rs766826	0.2397	0.3505	0.4715
rs61667602	0.06108	0.2252	0.07904
rs111837807	0.05307	0.06552	0.1045
rs77534576	0.003729	0.0271	0.04987
rs1405655	0.4866	0.3281	0.3753
rs67579710	0.2545	0.1069	0.07633
rs12809318	0.2765	0.4943	0.3757
rs35705950	0.01138	0.112	0.08843
rs721917	0.3946	0.4204	0.3276
rs117169628	0.01764	0.1436	0.09189
